# dysfunctional breathing and anxiety related disorder

**DOI:** 10.1192/j.eurpsy.2023.1433

**Published:** 2023-07-19

**Authors:** I. Sohn, I. Cho

**Affiliations:** 1 psychiatry, keyo hospital, Uiwang; 2 psychiatry, imom psychiatric clinic, seong-nam, Korea, Republic Of

## Abstract

**Introduction:**

Although dysfunctional breathing is a common symptom in general population and affects qualities of life, it is still underdiagnosed. There are some studies of prevalence of it in asthma, but few studies in mental illness.

**Objectives:**

The purposes of this study were to explore the prevalence of it in anxiety related disorders, and to investigate whether anxiety influence it.

**Methods:**

150 patients diagnosed with anxiety or depressive disorders, and 135 controls were recruited. Nijmegen questionnaire was used to assess dysfunctional breathing, and Hospital anxiety depression scale was used.

**Results:**

The prevalence of dysfunctional breathing in anxiety related disorders was higher than that in control.

In the linear regression model, anxiety accounted for 61.2 % of dysfunctional breathing, but depressed mood. With covariate adjusted for anxiety, scores of dysfunctional breathing in anxiety or depressive disorders were higher than in controls.

**Conclusions:**

Dysfunctional breathing in anxiety related disorders is higher than that in control. Adjusting anxiety, its difference is still. Anxiety affects dysfunctional breathing, but depressed mood does not.

**Disclosure of Interest:**

None Declared

